# Validation and psychometric evaluation of the Dutch person-centred care of older people with cognitive impairment in acute care (POPAC) scale

**DOI:** 10.1186/s12913-020-06048-x

**Published:** 2021-01-13

**Authors:** Annette Keuning-Plantinga, Evelyn J. Finnema, Wim Krijnen, David Edvardsson, Petrie F. Roodbol

**Affiliations:** 1 NHL Stenden University of Applied Sciences, Rengerslaan 8-10, Postbox 1298, 8900 CG Leeuwarden, The Netherlands; 2 Health Sciences-Nursing Science & Education University of Groningen & University Medical Center Groningen, Hanzeplein 1, Postbox 30.001, 9700 RB Groningen, The Netherlands; 3 University Medical Center Groningen, Groningen, The Netherlands; 4 Hanze University of Applied Sciences, Eyssoniusplein 18, 9714 CE Groningen, The Netherlands; 5 School of Nursing and Midwifery, La Trobe University, Level 4, Austin Tower, PO BOX 55555, Heidelberg, Victoria 3084 Australia; 6 Department of Nursing, Umeå University, 901 87 Umeå, Sweden

**Keywords:** Acute care, Dementia, Nurses, Person-centred care, Psychometrics, Quality of care

## Abstract

**Background:**

Person-centred care is the preferred model for caring for people with dementia. Knowledge of the level of person-centred care is essential for improving the quality of care for patients with dementia. The person-centred care of older people with cognitive impairment in acute care (POPAC) scale is a tool to determine the level of person-centred care. This study aimed to translate and validate the Dutch POPAC scale and evaluate its psychometric properties to enable international comparison of data and outcomes.

**Methods:**

After double-blinded forward and backward translations, a total of 159 nurses recruited from six hospitals (*n*=114) and via social media (*n*=45) completed the POPAC scale. By performing confirmatory factor analysis, construct validity was tested. Cronbach’s alpha scale was utilized to establish internal consistency.

**Results:**

The confirmatory factor analysis showed that the comparative fit index (0.89) was slightly lower than 0.9. The root mean square error of approximation (0.075, *p*=0.012, CI 0.057–0.092) and the standardized root mean square residual (0.063) were acceptable, with values less than 0.08. The findings revealed a three-dimensional structure. The factor loadings (0.69–0.77) indicated the items to be strongly associated with their respective factors. The results also indicated that deleting Item 5 improved the Cronbach’s alpha of the instrument as well as of the subscale ‘using cognitive assessments and care interventions’. Instead of deleting this item, we suggest rephrasing it into a positively worded item.

**Conclusions:**

Our findings suggest that the Dutch POPAC scale is sufficiently valid and reliable and can be utilized for assessing person-centred care in acute care hospitals. The study enables nurses to interpret and compare person-centred care levels in wards and hospital levels nationally and internationally. The results form an important basis for improving the quality of care and nurse-sensitive outcomes, such as preventing complications and hospital stay length.

**Supplementary Information:**

The online version contains supplementary material available at 10.1186/s12913-020-06048-x.

## Background

Worldwide, approximately 50 million people are living with dementia. Due to the ageing population, this number will increase [1]. People with dementia are regularly hospitalized due to comorbidities; they occupy approximately 25% of the hospital beds [2, 3]. This population is at risk for falls during a hospital stay, inadequate hydration and nutrition, delirium, infection, and functional decline [4–6]. These factors impact the duration of stay, the person’s functioning, and the care required following discharge [7]. Nursing care for people with dementia should be based on evidence, best-practice care, and processes combined with person-centred care to prevent complications [6, 8–11]. It is known that person-centred care can improve their quality of life. In spite of that, specific knowledge about person-centred care, also referred to as patient-centred or client-centred care, is limited in hospitals [11]. However, worldwide, it is the paragon in the care of people with dementia [10, 12]. The basis of person-centred care in caring for people with dementia is laid by Tom Kitwood [13, 14]. In a broader context, the framework of McCormack ad McCance is often used [15–17]. In the care for people with dementia, Brooker’s definition and framework are often used [3, 18].

To improve the quality of care for people with dementia in an acute care setting, knowledge of the level of person-centredness of the care is important. The literature reports a limited number of instruments that measure person-centred care for dementia patients in an acute hospital setting [8, 19]. Available instruments are aimed at long-term care [20] or more generically on person-centred care in the acute hospital setting and lack a specific focus on the quality of care for people with dementia [21–24].

The person-centred care of older people with cognitive impairment in acute care (POPAC) scale [19] was developed for the acute hospital setting and measures the person-centredness of care for older people with dementia. The scale consists of three subscales, which can be connected to the elements of person-centred care of the definition used. The subscale ‘using cognitive assessments and care interventions’ is suitable for valuing people; ‘using evidence and cognitive expertise’ is suitable for understanding situations from the perspective of the person with dementia; and ‘individualizing care’ is related to individualizing approaches and the social environment. In addition to measuring and improving the quality of care, translating tools into different language versions enables international comparisons of data and comparative analysis of levels, correlations, and person-centred care outcomes. In addition, there are no Dutch-language instruments available that measure person-centred care in the hospital setting.

The POPAC scale was designed in 2013 by Edvardsson in Australia to establish quantitative measurements to assess experienced levels of person-centred care for people with dementia in acute hospital settings [19, 25]. Based on the literature, eight dimensions of best practice were used to construct the instrument. Further development with a panel of international experts led to an instrument that consisted of statements on recognizing cognitive impairment, consulting specialist expertise, using evidence-based care protocols or guidelines, making environmental adjustments, providing social enrichments, prioritizing staff continuity and close interactions, avoiding restraints, and individualizing care [19]. The degree to which participants agree with item statements was expressed on a 6-point Likert scale with the categories ‘*never*’ [1], ‘*very rarely’* [2], ‘*rarely*’ [3], ‘*frequently*’ [4], ‘*very frequently’* [5], and ‘*always*’ [6] [10, 19, 25]. A significant Bartlett’s spherical test and a Kaiser-Meyer-Olkin (SME) sample adequacy measurement of > 0.7 were used to assess the construct validity. Construct validity was then assessed using principal component analysis with oblimin rotation due to the factors’ expected correlation [19].

The original instrument was pilot tested with a sample of 212 nurses from different types of wards, such as neurology, orthopaedics, and cardiology, in an acute care hospital in Melbourne, Australia. After the preliminary test, six items were removed because they did not meet the cutoff for acceptable homogeneity (> 0.3). A retest was conducted with 25 nurses from an orthopaedic ward, and the outcomes indicated satisfactory temporal stability [19]. The assumption that all items reliably measure a single underlying construct was supported by the item-total correlations ranging from 0.40 to 0.67, where values 0.4 and above indicate very good discrimination [26]. The subscales can be combined into a total score, where higher scores indicate higher person-centredness levels to evaluate the overall level of person-centred care. An interpretation of the score is not yet available. The totals of the items per subscale suggest possible areas for improvement of care. The instrument allows the comparison of person-centred care at both national and international levels [19].

Nilsson psychometrically evaluated the instrument in Sweden (2013), and Grealish (2017) evaluated the scale in Australia [10, 25]. Both Nilsson and Grealish used Cronbach’s alpha and corrected total correlation for internal consistency and CFA for construct validity. In addition, in Nilsson’s study, temporal stability was measured through the correlation between test and retest scores [10]. Both studies reported that the POPAC scale is valid and reliable and can be used to provide insight into nursing care’s person-centredness in a hospital setting. However, the high correlations between the subscales and the authors’ conclusion that the instrument’s dimensionality requires further research are important tenets for this study [10, 25]. For using the POPAC scale in the Netherlands to study person-centred care in a hospital setting, the instrument needed to be translated into Dutch. Measuring psychometric properties is important for assessing validity and reliability [27]. Nurses and nursing managers can use the outcomes of the POPAC scale to improve the quality of care in their ward, and outcomes and data can be used for national and international comparison. Therefore, this study aimed to conduct a cross-national validation and psychometric evaluation of the Dutch version of the POPAC scale.

## Methods

This study aimed to translate and validate the POPAC scale into Dutch and test the Dutch version of the questionnaires for psychometric properties among Dutch nurses working in acute hospital settings [28]. Data were collected with the online questionnaire program Qualtrics^XM^ (version 2018, Provo, UT USA).

### The instrument

The POPAC scale consists of 15 items, as shown in Table 1. The items describe care procedures and processes in patients with dementia in hospitals [19]. With the self-report of nurses in hospitals, the POPAC scale measures the extent to which nursing interventions are based on best practices in association with person-centred care. The items are divided into three subscales: cognitive assessments and care interventions (items 1–5), evidence and cognitive expertise (items 6–8), and individualizing care (items 9–15) [19]. The scores can be evaluated per subscale, or the score of the total scale can be used. The subscale or total scale scores can be calculated by dividing the sum of the scores by the number of items, whereby higher scores imply higher levels of person-centredness [10, 19, 25].

**Table 1:** Original items POPAC (Edvarsson, 2013) [19]

Item	
1	We assess the cognitive status of our older patients on admission
2	We make environmental adjustments to avoid over-stimulation in older people with cognitive impairment (e.g. single rooms, noise reductions etc.)
3	We diagnose symptoms of cognitive impairment (e.g. dementias, delirium etc.)
4	We spend more time with older patients with cognitive impairments as compared to cognitively intact patients
5	We leave older people with cognitive impairments alone in the ward
6	We use evidence-based tools to assess cognitive status of older patients (e.g. the MMSE, SPMSQ, CAM)
7	We consult specialist expertise (e.g. psychologist, gerontologist) if we find that a patient has cognitive impairment
8	We use evidence-based care guidelines in the care of older cognitively impaired patients
9	We use biographical information about older patients (e.g. habits, interests and wishes etc.) to plan their care
10	We involve family members in the care of older patients with cognitive impairment
11	We provide staff continuity for older patients with cognitive impairments (e.g. the same nurses providing care to these patients as often as possible)
12	We systematically evaluate whether or not older patients with cognitive impairment receive care that meets their needs
13	We involve older patients with cognitive impairment in decisions about their care (e.g. examinations, treatments etc.)
14	We ensure that older patients with cognitive impairment have tests/examinations/consultations in the unit rather than having to go to another department
15	We discuss ways to meet the complex care needs of people with cognitive impairment

### Translation of the person-centred care of older people with cognitive impairment on the acute care scale

The instrument’s principal author was involved in the translation, validation, and writing of the evaluation. Therefore, the instrument was translated according to the guidelines described by Sousa [29]. Two independent translators from a certified translation agency translated the questionnaire into Dutch. Two researchers (AK and EJF) independently assessed these two translations to determine the optimal translation of the question formulations and the answer options.

During the translation process, there was some discussion about using the term ‘cognitive functioning’ or ‘cognitive status’, whereby all translators agreed upon the choice for ‘cognitive functioning’ because this term is commonly used in nursing care in the Netherlands. There were no disagreements on a lingual or cultural basis. There was unanimous consensus for the final selection of all items.

This Dutch version was also translated back into English by two other independent translators from the same certified translation agency. These translations were again independently assessed by the same researchers to decide on the best translation. This time, there was consensus on all of the items. The author reviewed this final English version, and the conclusion was that the outcomes closely resembled the original version. There were no specific reasons to expect systematic errors during the translation due to linguistic or cultural differences [30]. The final version is attached as Additional file 1.

### Sample size

According to the scientific literature, the sample size depends on the number of factors and the factor load, where a minimum sample size of 100 is recommended, and a sample size of 150 is suggested for three-phase models [30, 31]. The COSMIN (Consensus-based Standards for the selection of health status measurement instruments) checklist advocates seven times the number of items [32]. Based on this knowledge, the optimal sample size was at least 150 [33, 34]. It may be noted that in the post hoc analysis, the sample size was sufficient for almost all estimated parameters to be (highly) significant.

### Setting, recruitment, and participants

Six hospitals in the northern part of the Netherlands participated in this study and were supplemented by Dutch nurses who were recruited via LinkedIn and Facebook. The data were collected in one university hospital, two non-university teaching hospitals, and three rural hospitals. The capacity of the hospitals varied from 241 to 1300 beds, with additional outpatients.

Nurses with at least three months of experience in the clinical setting, working in the direct care of people with dementia, and willing to participate were included in the study. All hospital departments were included, except for paediatrics and obstetrics. The data collection took place from July 2018 to March 2019.

The recruitment of participants in the hospitals was performed by contact persons working in the hospital based on a convenience sample [27]. The authors also used LinkedIn and Facebook to recruit hospital nurses. A general request was made for nurses to participate via LinkedIn, in which nursing managers are active and then the call was repeated once. For Facebook, which is often used by Dutch nurses, a different approach was used for which the authors requested two groups on Facebook. One was in a private group for questionnaires of a professional nursing magazine, and the other was in an open group for nurses in general. On Facebook, a daily update of the response was provided. This Facebook group has many members; however, it is not known how many members are active.

### Data analysis

For the data analysis, we used IBM SPSS statistics (for Macintosh, version 25, Armonk, NY: IBM Corp.). Only complete scales were used in the data analysis. To perform confirmatory factor analysis (CFA), JASP (Version 0.11.1) with Lavaan was used [33]. Before starting the analysis, Item 5 was reverse coded due to the negative wording of this item. The decision to use only completed scales was made based on the response rate of 159 complete cases instead of 164 with the inclusion of incomplete scales. Because the sample was sufficiently large and the differences in outcomes were minimal, it was decided that only completed questionnaires would be included. This makes the data as accurate as possible.

The Shapiro-Wilk test was used to assess the normality of the distribution. Descriptive analyses were used to describe the sample. Item performance was assessed by calculating item means and standard deviations, the inter-item correlation matrix, and the corrected item-total correlation.

The CFA was performed by robust maximum likelihood estimation, after which four types of fit indices were used to evaluate the fit of the model to the data: the chi-square model fit, the comparative fit index (CFI), the root mean square error of approximation (RMSEA), and the standardized root mean-square residual (SRMR). The Hoelter index was utilized to check the smallest sample size at which the chi-square interpretation would not be significant. As a criterion for significance, a *p*-value < 0.05 was used. The model fit was considered acceptable if the following criteria were met: p-value for the χ2 model fit compared to the baseline model smaller than 0.05, CFI and GFI values between 0.90 and 0.95 or above RMSEA and RMR values of 0.08 or below [34].

Cronbach’s alpha on the total scale and its subscales were assessed to determine the internal consistency.

### Ethics approval and consent to participate

The study was performed following the Helsinki declaration, and all of the participants provided written informed consent before filling out the questionnaire. Nurses had an option to choose whether the results would also be available for further research. The Medical Ethical Committee of the University Medical Center Groningen considered approval unnecessary (decision M17.221048) because the questionnaire was intended for staff. The questionnaire was completely anonymous; no one could be identified based on the results. The managers received an email with a general explanation and a link to the questionnaire to forward it to the nurses of their team. Managers were not informed about the number of participating nurses from their ward or about their responses. Based on the contact persons’ information and the response per ward, there was no reason to believe that nurses felt obliged to participate in this survey. The voluntary nature of participation was emphasized in the explanations.

## Results

### Characteristics of the sample

In total, 159 hospital nurses completed the POPAC scale; 114 nurses were recruited directly from hospitals, and 45 nurses were recruited through social media. The hospitals’ general response rate was 33%, based on the managers of the participating wards’ information. More specifically, responses came from nurses working in medical (21.4%), surgical (20.1%), and geriatric (13.2%) wards as well as in wards with different combinations of specialized care (45.3%), as shown in Table 2. The education of the nurses varied from a care assistant level to a master level. The nurses had an average experience of 18 years of working with the elderly population, ranging from a few weeks to 45 years (SD 12.6). A total of 43.3% of the nurses had participated in a course in the past year about care for people with dementia. They graded their skills on caring for people with dementia with an average of 7.3 on a scale from 1 to 10 with a range from 4 to 9 (SD 0.095).

**Table 2:** Characteristics of nurses (n=159)

		Directly from hospital		Via Social Media		Total	
		Frequency	Percent	Frequency	Percent	Frequency	Percent
Level of nurses	Student level	0	0	1	2.2	1	0.6
	Care assistant	1	0.9	1	2.2	2	1.3
	Secondary vocational level	60	53.5	24	53.3	64	52.8
	Bachelor level	52	46.6	8	17.8	60	37.7
	Master level	1	0.9	11	24.4	12	7.6
Ward type	Medical	17	14.9	17	37.8	34	21.4
	Surgical	23	20.2	9	20	32	20.1
	Geriatric	17	14.9	4	8.9	21	13.2
	Other	57	50	15	33.3	72	45.3
		**Mean**	**SD**	**Mean**	**SD**	**Mean**	**SD**
Years’ experience in working with elderly		19.7	12,7	13,7	11.15	18	12.6
Grade skills		7.1	1,5	7.2	1.1	7.2	1.8
Followed a course on dementia last year	Yes	50	43.8	19	42.2	69	43.4
	No	64	56.1	26	57.8	90	56.6

### Item performance

The mean score per item varied between 3.59 and 5.28, as shown in Table 3. The total score was 66.88 (SD 10.04), with a mean of 4.46 (SD 0.53). The Shapiro-Wilk test indicated that the data were skewed. The skewness per item varied between − 0.04 and − 1.83. Internal consistency was based on a cutoff point of Cronbach’s alpha 0.7, an item-total correlation of 0.3, and inter-item correlations between 0.2–0.4 [26]. The correlation between the different items revealed some negative correlations with Item 5. It shows a corrected item-total correlation of 0.11. The other values varied from 0.34 (Item 14) to 0.63 (Items 8 and 9). The Cronbach’s alpha increased by 0.1 when Item 5 was deleted. The visual expectation of the data gave indications for a three-block structure.

**Table 3:** Median (SD), Mean (SD), Item-rest correlation, Item-total correlation, Cronbach’s alpha if item deleted, Cronbach’s alpha overall, and Cronbach’s alpha per subscale (*n*=159)

	Median	SD^a^	IQR^b^	Mean	SD^a^	Item-rest correlation	Item-total correlation	Cronbach’s alpha if item deleted	Cronbach’s alpha overall	Cronbach’s alpha per subscale
Item1	6	1.05	1	5.28	1.06	0.48	0.54	0.84	0.85	**1. Using cognitive assessments and care interventions** with item 5: 0.60 without item 5: 0.72
Item2	5	1.05	1	4.75	1.05	0.56	0.67	0.84		
Item3	5	1.06	2	4.90	1.06	0.46	0.49	0.84		
Item4	4	1.20	2	3.87	1.20	0.43	0.48	0.84		
Item5	5	1.31	2	4.64	1.31	0.11	−0.32	0.86		
Item6	6	1.32	1	5.09	1.32	0.55	0.59	0.84		**2. Using evidence and cognitive expertise** 0.78
Item7	5	1.05	1	5.06	1.05	0.48	0.51	0.84		
Item8	5	1.13	1	4.45	1.13	0.63	0.67	0.83		
Item9	5	1.07	2	4.27	1.07	0.63	0.64	0.83		**3. Individualizing care** 0.80
Item10	5	0.88	1	4.77	0.88	0.48	0.51	0.84		
Item11	4	1.29	2	3.62	1.29	0.52	0.51	0.84		
Item12	4	1.37	2	3.59	1.37	0.59	0.58	0.83		
Item13	4	1.12	2	4.16	1.12	0.34	0.34	0.85		
Item14	4	1.46	3	4.26	1.46	0.35	0.37	0.85		
Item15	4	1.18	2	4.15	1.18	0.73	0.73	0.83		

^a^Standard Deviation

^b^Interquartile Range

### Construct validity

Construct validity was evaluated with the CFA loadings for the items and the interfactor correlations based on ML estimation. Lavaan’s iterative maximum likelihood estimation converged after 22 iterations. An overview of the different fit indices is shown in Table 4 and indicates an acceptable model fit. The Hoelter’s critical N was 106.8, which means that the sample size was adequate.

**Table 4:** Fit indices

Metric	Value
Comparative Fit Index (CFI)	0.89
Root mean square error of approximation (RMSEA)	0.08
RMSEA 90% CI lower bound	0.06
RMSEA 90% CI upper bound	0.09
RMSEA p-value	0.01
Standardized root mean square residual (SRMR)	0.06
Hoelter’s critical N (α = .05)	106.88
Goodness of fit index (GFI)	0.99
Expected cross validation index (ECVI)	1.64

A chi-square test was performed to check the model fit. This test showed that the factor model differed significantly from the baseline model, χ2 (87, *N*=159) =164,84, *p*<.001. The obtained CFI of 0.89 was slightly smaller than the cutoff value of 0.9. Both the RMSEA (0.075, *p*=0.012, CI 0.057–0.092) and the SRMR of 0.063 were acceptable, with values less than 0.08.

The CFA showed that all loadings were fairly large, positive, and significantly different from zero, as presented in Table 5 with Item 5 as the only exception. The factor correlations were between 0.69 and 0.77, indicating that the factors were strongly associated.

**Table 5:** Factor loadings

Factor loadings								
							95% Confidence Interval	
Factor	Indicator	Symbol	Estimate	Std. Error	z-value	p	Lower	Upper
Factor 1	Item1	λ11	0.675	0.117	5.754	< .001	0.445	0.905
	Item2	λ12	0.711	0.095	7.465	< .001	0.524	0.898
	Item3	λ13	0.649	0.094	6.944	< .001	0.466	0.833
	Item4	λ14	0.641	0.106	6.058	< .001	0.434	0.849
	Item5	λ15	0.057	0.106	0.538	0.590	−0.150	0.264
Factor 2	Item6	λ21	1.000	0.127	7.878	< .001	0.751	1.249
	Item7	λ22	0.671	0.098	6.814	< .001	0.478	0.864
	Item8	λ23	0.899	0.098	9.176	< .001	0.707	1.091
Factor 3	Item9	λ31	0.756	0.078	9.706	< .001	0.603	0.909
	Item10	λ32	0.496	0.069	7.171	< .001	0.360	0.631
	Item11	λ33	0.753	0.099	7.589	< .001	0.559	0.948
	Item12	λ34	0.930	0.092	10.120	< .001	0.750	1.110
	Item13	λ35	0.501	0.085	5.891	< .001	0.334	0.668
	Item14	λ36	0.606	0.127	4.790	< .001	0.358	0.854
	Item15	λ37	0.948	0.078	12.198	< .001	0.796	1.100

**Fig. 1:**
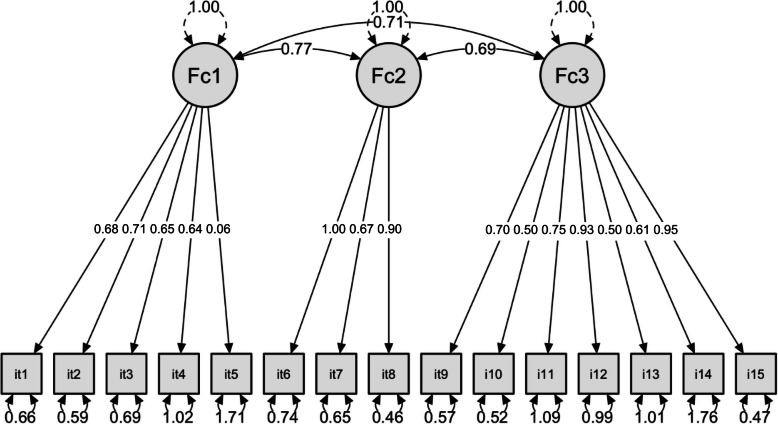
Model with factor loadings, residual variances, and factor covariances (*n*=159)

Figure 1 provides the final model with significant correlations between the subscales, residual variances, and factor covariances.

### Internal consistency

For measuring internal consistency, Item 5 was reversed. The total instrument’s internal consistency measured by Cronbach’s alpha was 0.85 (CI 0.82–0.88). The internal consistency of using cognitive assessments and care interventions was 0.60 (CI 0.45–0.66) with item five and 0.72 (CI 0.63–0.78) without it; using evidence and cognitive expertise had an internal consistency of 0.78 (CI 0.70–0.83) and individualizing care 0.8 (CI 0.74–0.84).

## Discussion

This study aimed to translate and validate the Dutch version of the POPAC scale and evaluate the psychometric properties to make international comparisons possible. The outcomes confirm that this Dutch version of the POPAC scale is a valid and reliable instrument for measuring person-centred care and the quality of care of people with dementia in acute care [10, 19, 25].

The results obtained from the factor analysis with three factors were comparable with those from earlier research [10, 19, 25]. All of the earlier studies derived a three-factor solution whereby Nilsson found that Cronbach’s alpha values of Subscales 2 (using evidence and cognitive expertise) and 3 (using evidence and cognitive expertise) did not reach the necessary cutoff point of 0.7 [10]. Grealish used an exploratory factor analysis because the items did not meet the predetermined cutoff points for using confirmatory factor analysis [25]. They created a revised version of the instrument in which Item 5, concerning leaving people with cognitive impairments alone in the ward, was deleted, and several items were grouped into another subscale. The model fit confirmed the three-factor solution. That is, the Chi-square rejected the model. However, this test has been found to be unreliable for small sample sizes [35]. The CFI indicated a nearly acceptable model fit, as with Nilsson and Grealish, who reported CFIs of 0.88 and 0.90, respectively [10, 25]. The RMSEA and the SRMR suggested an acceptable model fit [35]. However, the findings confirmed the three-dimensional structure suggested by previous research. The loadings of the items indicate that these are strong associations with each of the factors. In addition, the factor correlations also indicated that there were strong associations, which indicated that the factors were strongly associated with one general factor of the POPAC scale. Future research is necessary to elucidate the scientific benefits of distinguishing three factors in explaining person-centred care over that of a single generic POPAC factor. The Dutch version of the POPAC scale has similar results as the Edvardsson and Nilsson study [10, 19]. Grealish assigned three variables to other subscales [25]. In the current study, evidence was found that the POPAC has psychometric properties very similar to those previously reported in the literature. For this reason, the POPAC can be applied in the Netherlands as three separate subscales as well as a total scale measuring the level of person-centred care.

Furthermore, Cronbach’s alpha of 0.86 corresponds with earlier research in which the internal consistency varies from 0.83 to 0.87 [10, 19, 25]. Additionally, this research confirms that, statistically, Item 5 (about leaving patients with cognitive impairments alone), which is on the ‘Using cognitive assessments and care interventions’ subscale, could be deleted to improve the instrument’s internal consistency. This is because this subscale has an internal consistency of 0.6, which is lower than the cutoff of 0.7 [26, 27]. Instead of deleting this item, the authors suggest rephrasing it into a positively worded item. It is the only negatively formulated item, which may influence the outcomes. The background of the instrument’s construction can provide direction in changing the focus of this question. Nurses are always present in the hospital ward, so they do not experience leaving patients by themselves. However, this does not mean that people with dementia are always visible to nurses and monitored when needed, which might influence care. Our suggestion is to reformulate this question from:

“*We leave older people with cognitive impairments alone in the ward.”*

to:

*“We make sure older people with cognitive impairments are not left alone in the ward*.”

The mean scores in our study were higher than the scores in earlier studies. A higher score reflects a higher level of the construct of person-centred care [19]. This score can partly be explained by the obtained high score found for Item 1 regarding assessing cognitive status. In the Netherlands, assessing cognition is a criterion on which hospital care quality is judged, which might have influenced this positive outcome.

Scores on the POPAC scale among a sample of nurses can be utilized to measure the level of person-centred care for people with dementia in hospitals. Nursing professionals and nursing managers can use the outcomes as indicators to determine which areas of care can be improved in their ward [36]. Additionally, the POPAC scale can be used in the education of nurses and nursing students to create awareness of person-centred care. The POPAC scale is applicable in research on person-centred care, for example, to investigate if a relationship exists between the outcomes of the POPAC scale and complications such as falls, poor hydration and nutrition, delirium, infection, and functional decline. It can also be used to determine whether there is a relation between the level of person-centredness of the care and the length of the hospital stay. In brief, the POPAC scale can be applied to investigate various important research questions regarding interventions for people with cognitive impairment in acute wards. The authors will use this instrument to determine nurses’ perceptions of person-centred care for people with dementia.

### Limitations

The POPAC scale is a relatively novel instrument, and its validity and reliability need to be further developed. There is no gold standard available to compare the results with. This study aimed to measure the validity by using factor analysis, as in previous studies. This was done using one group. To improve construct validity, the authors suggest using other methods to strengthen the theoretical basis, such as item response theory, the use of multiple groups, and a test-retest construction.

Our study had a lower response rate (33%) in the hospital setting than those of previous studies, e.g., 59% [19], 51% [10], and 54.3% [25], possibly due to the different methods of recruiting responders. There were two primary aspects. On the one hand, nursing managers did not always want to cooperate because there were only a small number of people with dementia in their ward, or they perceived no added value in the study. This could result in a nonresponse bias and affect the external validity of the study. The nonresponse may have caused some bias in the direction of the null. However, since our results were mainly in line with those previously found in the literature, we consider this bias to be relatively weak.

By using a convenience sample, participating nurses with a high affinity for the topic may be overrepresented. This leads to a limitation of the external validity and, consequently, the generalization. Since the recruitment was rather general, yielding a rather broad sample of participants, we expect the results to be generalizable for the setting of general hospitals. In future research, it may be useful to validate actual care provision and behaviour in practical working situations. However, the combination of nurses from hospitals and via social media provided a significant scope of the Netherlands.

The questionnaire was conducted in combination with another lengthy questionnaire. The numerous questions negatively influenced the motivations to complete the questionnaire, which could have caused missing information. This may affect internal validity negatively. We used different orders of the questionnaire to prevent this bias.

## Conclusions

The findings of this study confirm the validity and reliability of the Dutch version of the POPAC scale. However, the results provide grounds for further research on the instrument’s dimensionality with a rephrased Item 5. The results can help nursing managers improve person-centred care in hospitals for people with dementia. The authors advise using total sum scores for interpretation of the scale for national and international comparison. Further research can provide insight into the relationship of person-centred care with the quality of care and nurse-sensitive outcomes, such as preventing complications hospital stay length.

### Implications for nursing practice

The results are of significance for nurses in facilitating the improvement of care for people with dementia. The instrument can be used to hold reflective discussions in clinical settings about the extent to which nurses can perform person-centred care and how they can improve this care. This study’s findings also enable the broader use of the POPAC scale: a total sum score can be calculated and consequently used to determine and interpret the level of person-centred care. Person-centred care and evidence-based nursing are important ingredients for high-quality nursing care for people with cognitive impairments. Therefore, the instrument is easy for nurses to use as an instrument for practice improvement. Furthermore, nurses can employ the results of the POPAC scale for benchmarking the level of person-centred care at a hospital as well as on a national or international level.

## Supplementary Information

**Additional file 1.** Dutch version of the POPAC scale.

## Data Availability

The datasets generated and analysed during the current study are not publicly available, as more papers will be written based on this dataset. The corresponding author can provide data upon reasonable request.

## Abbreviations

CFA: Confirmatory Factor Analysis
CFI: Comparative Fit Index
CI: Confidence interval
COSMIN: Consensus-based Standards for the selection of health status Measurement Instruments
POPAC: Person-centred care of Older People with cognitive impairment in Acute Care
RMSEA: Root Mean Square Error of Approximation
SD: Standard deviation
SRMR: Standardized Root Mean-square Residual
