# Supplementary material for: Validation and psychometric evaluation of the Dutch person-centred care of older people with cognitive impairment in acute care (POPAC) scale
Source: BMC Health Serv Res. 2021 Jan 13;21:59. doi: 10.1186/s12913-020-06048-x (PMC7805135; doi:10.1186/s12913-020-06048-x)
Supplement: Supplementary file 1 — Additional file 1. Dutch version of the POPAC scale. [file 12913_2020_6048_MOESM1_ESM.docx]

**Additional file 1. Dutch version of the POPAC**

POPAC-NE

| 1 | We beoordelen de cognitieve status van onze oudere patiënten bij opname. |
| --- | --- |
| 2 | We passen de omgeving aan om prikkels te vermijden bij ouderen met cognitieve stoornissen (bijvoorbeeld eenpersoonskamers, lawaaibeperking). |
| 3 | We diagnosticeren symptomen van cognitieve stoornissen (bijvoorbeeld dementie, delier, etc.). |
| 4 | We brengen meer tijd door met oudere patiënten met cognitieve stoornissen in vergelijking met patiënten zonder cognitieve problemen. |
| 5 | We gebruiken evidence-based hulpmiddelen om de cognitieve status van oudere patiënten te beoordelen (bijvoorbeeld de MMSE, SPMSQ, CAM, DOS). |
| 6 | We raadplegen specialistische expertise (bijvoorbeeld psycholoog, gerontoloog) als we vaststellen dat een patiënt cognitieve beperkingen heeft. |
| 7 | We gebruiken evidence-based zorgrichtlijnen bij de zorg voor oudere cognitief beperkte patiënten. |
| 8 | We gebruiken persoonlijke informatie over oudere patiënten (bijvoorbeeld gewoonten, interesses en wensen etc.) om hun zorg te plannen. |
| 9 | We betrekken familieleden bij de zorg voor oudere patiënten met cognitieve stoornissen. |
| 10 | We bieden personeelscontinuïteit voor oudere patiënten met cognitieve stoornissen (bijvoorbeeld dezelfde verpleegkundigen die zo vaak mogelijk zorg verlenen aan deze patiënten). |
| 11 | We evalueren systematisch of oudere patiënten met cognitieve stoornissen al dan niet zorg ontvangen die aan hun behoeften voldoet. |
| 12 | We betrekken oudere patiënten met cognitieve stoornissen bij beslissingen over hun zorg (bijvoorbeeld onderzoeken, behandelingen enz.). |
| 13 | We zorgen ervoor dat oudere patiënten met cognitieve stoornissen testen/ onderzoeken/consulten op de afdeling hebben in plaats van naar een andere afdeling te moeten gaan. |
| 14 | We bespreken met elkaar manieren om tegemoet te komen aan de complexe zorgbehoeften van mensen met cognitieve stoornissen. |
| 15 | We beoordelen de cognitieve status van onze oudere patiënten bij opname. |
